# Systematic Understanding of Pathophysiological Mechanisms of Oxidative Stress-Related Conditions—Diabetes Mellitus, Cardiovascular Diseases, and Ischemia–Reperfusion Injury

**DOI:** 10.3389/fcvm.2021.649785

**Published:** 2021-04-13

**Authors:** Mengxue Wang, Yun Liu, Yin Liang, Keiji Naruse, Ken Takahashi

**Affiliations:** Department of Cardiovascular Physiology, Graduate School of Medicine, Dentistry and Pharmaceutical Sciences, Okayama University, Okayama, Japan

**Keywords:** oxidative stress, reactive oxygen species, inflammation, diabetes mellitus, ischemia–reperfusion injury, mitochondria, transient receptor potential channels

## Abstract

Reactive oxygen species (ROS) plays a role in intracellular signal transduction under physiological conditions while also playing an essential role in diseases such as hypertension, ischemic heart disease, and diabetes, as well as in the process of aging. The influence of ROS has some influence on the frequent occurrence of cardiovascular diseases (CVD) in diabetic patients. In this review, we considered the pathophysiological relationship between diabetes and CVD from the perspective of ROS. In addition, considering organ damage due to ROS elevation during ischemia–reperfusion, we discussed heart and lung injuries. Furthermore, we have focused on the transient receptor potential (TRP) channels and L-type calcium channels as molecular targets for ROS in ROS-induced tissue damages and have discussed about the pathophysiological mechanism of the injury.

## Introduction

At first glance, diabetes, which causes abnormal blood glucose control, and ischemia–reperfusion injury (IRI) of the heart, which causes myocardial infarction, seem to have nothing in common. However, both these diseases are consistent in that they cause inflammation with the release of cytokines and the responses of immune cells. These reactions are triggered by the oxidative stress (OS) that occurs in the body. Oxidative stress is defined as an imbalance between oxidants and anti-oxidants in favor of the oxidants (1). Reactive oxygen species (ROS) including hydrogen peroxide (H_2_O_2_) and superoxide (^.^${\text{O}}_{2}^{-}$) that are generated in the cells cause OS when they become excessive. Oxidative stress causes diseases such as diabetes (2), IRI (3), cancer (4), and Alzheimer's disease (5), and, notably, this condition is affected by diet and obesity (6).

While the organ heart has drawn much attention in the context of ischemic heart diseases, which is the leading cause of death among humans (7), IRI also occurs in several other organs such as the lung (8). In addition, transplantation of organs, such as lungs and kidneys, can result in IRI due to blood reperfusion in ischemic-isolated organs (9). While having their own specific mechanisms for the development of diseases, the pathological conditions of diabetes and IRI also share a common molecular basis in a series of intracellular signal transduction mechanisms originating from OS, as discussed in the present review. In addition to diabetes, extending the pathophysiology of IRI from the perspective of OS is meaningful to understand the diseases and development of preventive measures and treatments involved.

## Pathophysiological Relationship Between Diabetes and Cardiovascular Diseases From the Perspective of ROS

As the life-expectancy of diabetic patients has increased significantly, the cardiovascular complications of diabetes have become prominent. When compared with people without diabetes, people with type 2 diabetes (T2DM) are at an increased risk of cardiovascular diseases (CVD) (10). The increased production of ROS in the diabetic heart is an important factor in the occurrence and development of diabetic cardiomyopathy (11). Reactive oxygen species can induce the inactivation of the signaling mechanism between the insulin receptor and the glucose transport system, which can lead to insulin resistance (12). Meanwhile, diabetes is a producer of OS, which can lead to atherosclerosis (13, 14). We have explored the mechanisms by which T2DM triggers OS and increases the risk of CVD from the prospect of obesity, hyperglycemia, and intracellular calcium.

### Obesity Plays an Important Role in Heart Disease of Diabetic Patients

A recent study reported presence of differences in the factors causing OS in the hearts of obese and non-obese diabetic mice. In addition, the decreased expression of antioxidant molecules in the hearts of non-obese diabetic mice was reported to act as an important factor that leads to the development of heart diseases (15). In this study, Li et al. created two groups of T2DM mouse models: obese and non-obese groups. They found that obese T2DM mice demonstrated more severe heart remodeling and earlier contractile dysfunction than non-obese T2DM mice. In addition, obese T2DM mice revealed severe and persistent myocardial lipotoxicity, which was manifested by increased free fatty acids (FFA) uptake. Excessive FFA uptake activates the peroxisome proliferator-activated receptor alpha (PPARα) pathway and phosphorylate glycogen synthase kinase 3 beta (GSK-3β), while inhibiting glucose transporter 4 (GLUT4) and fatty triglyceride lipase (ATGL). Among the tissue damage caused by lipotoxicity, OS is the main factor (16). Under the effect of lipotoxicity, the tissues absorb a large amount of FFA, leading to excessive oxidation of FFA, a sharp increase in the amount of oxygen consumption, and excessive ROS production (17–20). In addition, excessive FFA and resultant oxidation lead to ceramide synthesis, which in turn leads to increased cardiomyocyte apoptosis through the mitochondrial pathway (20).

Another interesting mechanism by which obesity affects the development of atherosclerosis through OS is Na/K-ATPase. According to Krithika Srikanthan et al., activation of the Na/K-ATPase signal cascade exacerbates obesity, diabetes, dyslipidemia, and atherosclerosis, and these conditions are all related to the imbalance of OS (21). Na/K-ATPase is a scaffold and signaling protein, and is also involved in many clinical conditions, including CVD and chronic kidney disease (22, 23). Fat accumulation in humans and mice is related to systemic OS (24). The white adipose tissue of obese mice has a trend of increased expression of NADPH oxidase (NOX) and decreased expression of antioxidant enzymes (25, 26). In cultured adipocytes, the production of ROS was significantly increased during the differentiation of 3T3-L1 cells into adipocytes, indicating that the production of ROS increased simultaneously with the accumulation of fat in adipocytes (27). Besides, the increase in free fatty acid levels can induce ROS production through the activation of NOX (28). Furthermore, diet-induced OS can activate the Na/K-ATPase/Src/ROS amplification loop, leading to the occurrence and development of dyslipidemia and atherosclerosis (21).

The nuclear factor erythroid 2-related factor 2 (NRF2) pathway is closely related to antioxidant effects and is activated at the onset of OS (29). Li et al. reported that the expression level of NRF2 and its target genes heme oxygenase 1 (HO-1) and NAD(P)H quinone dehydrogenase 1 (NQO1) increased significantly in the heart of obese T2DM mice, but they decreased in the hearts of non-obese T2DM mice (15). This result implies that myocardial lipotoxicity and antioxidant pathway activation occur in obese T2DM patients. This finding may provide a new guidance for the prevention and clinical treatment of diabetic heart diseases.

### Relationship Between Increased ROS Caused by Hyperglycemia and Cardiovascular Dysfunction

Hyperglycemia (high levels of blood glucose) leads to increased production of ROS, which ultimately leads to vascular dysfunction (30). Meanwhile, OS from hyperglycemia leads to insufficient glucose uptake by muscles and fat cells. Furthermore, OS from hyperglycemia may promote β-cell dysfunction and reduce insulin secretion by β cells (13, 31). This event also leads to further aggravation of hyperglycemia. As a result, hyperglycemia and OS interact. It is therefore important to understand how to reduce OS so as to reduce hyperglycemia.

**Figure 1:**
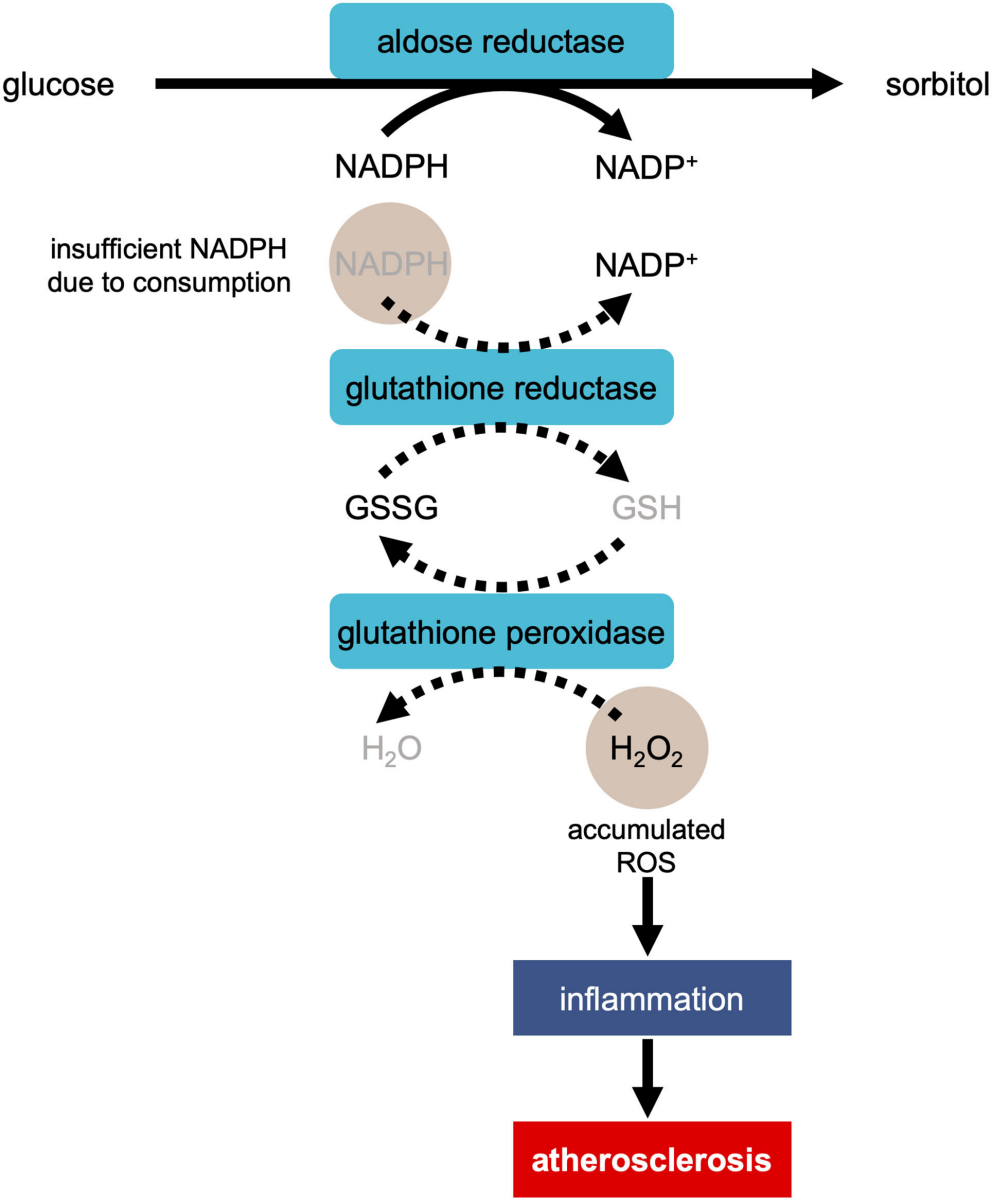
Development of atherosclerosis via ROS production in the polyol pathway in the condition of hyperglycemia. In the process of the reduction of glucose to sorbitol by aldose reductase, NADPH is oxidized to NADP^+^, consuming NADPH. As NADPH is essential for regeneration of antioxidant glutathione (GSH), the reaction of reducing H_2_O_2_ to H_2_O is suppressed. The accumulation of H_2_O_2_ causes inflammation, resulting in the development of atherosclerosis. GSSG, glutathione disulfide.

Another question that needs resolution is how does high blood sugar level trigger OS and lead to cardiovascular dysfunction. Under a hyperglycemic condition, ROS accumulates, damages DNA and proteins, and injures cardiomyocytes. The increase in ROS production caused by hyperglycemia occurs through the following ways: activation of the protein kinase C (PKC) pathway via diacylglycerol (DAG), increased hexosamine pathway flux, increased production of advanced glycation-end product, and increased flux in the polyol pathway (32, 33). During the ROS production in the polyol pathway, when aldose reductase reduces glucose to sorbitol, excess glucose enters the polyol pathway (Figure 1) (34). This reaction oxidizes NADPH to NADP^+^, consuming NADPH (34). As NADPH is essential for antioxidant regeneration, the decrease in the amount of NAPDH leads to the facilitation of OS.

Simultaneously, the accumulation of ROS caused by hyperglycemia triggers insulin resistance (13, 35, 36). Insulin resistance occurs when the cells in the muscles, fat, and liver do not respond appropriately to insulin and cannot uptake glucose from the blood for deriving energy (37). In response, the pancreas produce more insulin (37). Interestingly, insulin resistance is a component of T2DM, high blood pressure, and dyslipidemia; these characteristics together constitute a major risk of CVD (38).

Past studies have reported that mitochondrial OS is related to insulin resistance (39). Therefore, under high blood sugar level conditions, the mitochondria are active and produce more ROS (40). Elevated ROS levels can induce mitochondrial division, which in turn affects the insulin-PI3K-AKT pathway and GLUT4 (12). Glucose transporter 4 is the main glucose transporter (41) in the skeletal muscles and adipose tissue. The cells respond to insulin by increasing the expression of GLUT4 in the plasma membrane, thereby increasing the cellular uptake of blood glucose. When the glucose level is high, the body produces insulin, which then activates the PI3K/AKT pathway (42). Mitochondrial fission is directly related to insulin resistance of the skeletal muscles (43). Past studies have also demonstrated that restricting mitochondrial overactivation can prevent insulin resistance (44). In addition, insulin resistance caused by mitochondrial dysfunction may lead to metabolic and cardiovascular abnormalities, thereby increasing the incidence of CVD (38, 45). In summary, OS caused by hyperglycemia plays an important role in cardiovascular dysfunction and both the conditions interact with and influence each other.

### Effect of OS on Calcium Handling in the Heart Under Diabetic Conditions

Redox regulation of calcium-handling proteins directly affects cardiac contraction by changing intracellular calcium concentration (46). As discussed earlier, hyperglycemia in the cells can lead to excessive ROS production. The increase in the ROS level can inhibit autonomic ganglion synaptic transmission by oxidizing the α3 subunit of nicotinic acetylcholine receptor, which may in turn result in fatal arrhythmia (47). At the same time, ROS leads to sudden death of a diabetic patient after myocardial infarction by increasing post-translational protein modification, which leads to the downregulation of Ca^2+^-ATPase transcription in the sarcoplasmic reticulum.

Ventricular contraction and relaxation are mainly controlled by the release and uptake of Ca^2+^ by the sarcoplasmic reticulum Ca^2+^-ATPase 2 (SERCA2) pump (48, 49). In hypertrophic and failing myocardium, the level of SERCA2 protein and its ability to absorb Ca^2+^ are inhibited. Reactive oxygen species can oxidize and directly enhance CaMKII activity, which in turn phosphorylates and activates several Ca^2+^-handling proteins such as the cardiac ryanodine receptor RyR2 or cardiac SERCA (50).

**Figure 2:**
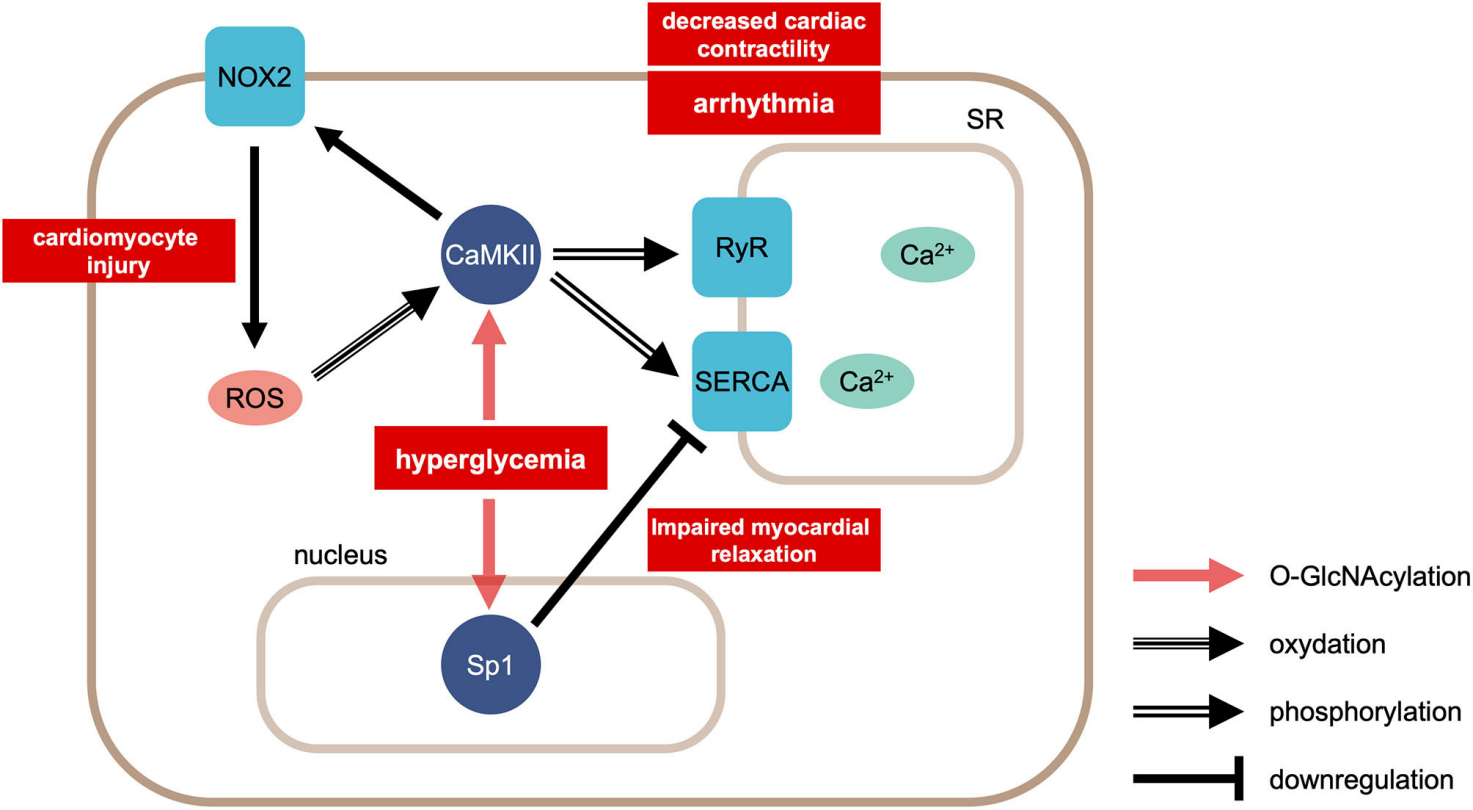
Calcium handling in cardiomyocytes in the condition of hyperglycemia. Hyperglycemia causes modification of CaMKII by O-linked N-acetylgulcosamine (O-GlcNAcylation). This modification facilitates ROS production via NOX2. ROS enhances CaMKII activity by oxidation. CaMKII phosphorylates RyR2 and SERCA. On the other hand, hyperglycemia induces GlcNAcylation of the transcription factor Sp1, reducing the transcription of SERCA2. CaMKII, Ca^2+^/calmodulin-dependent protein kinase II; ROS, reactive oxygen species; NOX2, NADPH oxidase 2; RyR2, ryanodine receptor 2; SERCA, sarcoplasmic reticulum Ca^2+^-ATPase 2.

Protein O-linked-N-acetylglucosaminylation (O-GlcNAcylation) plays important roles in calcium handling under diabetic conditions (Figure 2). For example, hyperglycemia increases the O-GlcNAc modification of calcium/calmodulin-dependent protein kinase IIδ (CaMKIIδ), which in turn leads to the autonomous activation of CaMKII (51, 52). Furthermore, the hyperglycemia-induced O-GlcNAcylation of CaMKII causes ROS production by NOX2 (53). Autonomous activation of CaMKII can lead to decreased cardiac contractility and potential fatal arrhythmias, such as ventricular premature beats and delayed depolarization. In fact, delayed depolarization is related to long QT interval arrhythmia (54). On the other hand, in the chronic hyperglycemia condition in diabetes, O-GlcNAc transferase reduces the transcription of SERCA2, which results in decreased calcium reuptake and impaired relaxation (55). The overexpression of GlcNAcase or the inhibition of GlcNAc modification increases the expression of SERCA2a, the ablated sarcoplasmic reticulum Ca^2+^ leakage, improved cardiac contractility, and reduced arrhythmia events (56).

In summary, calcium plays an important role in cardiac dysfunction caused by ROS derived under the condition of hyperglycemia.

## IRI in Terms of Oxidative Damage

ischemia–reperfusion injury is a type of tissue damage that occurs when the blood flows back to the tissue after a period of ischemia or under the lack of oxygen. IRI is often detected in cases of organ transplants, major organ resections, and shock. The main organs in which IRI occurs are the heart, lung, brain, liver, kidney, and intestine (57–62). This finding contributes to morbidity and mortality occurring in a variety of pathologies, such as myocardial infarction and stroke caused by coronary atherosclerosis (63).

ischemia–reperfusion is often associated with microvascular injury, especially due to increased permeability of the capillaries and arterioles, which lead to increased interstitial diffusion and fluid filtration across the tissues. After ischemia, the re-entry of blood into the tissue induces the release of large amounts of oxygen free radicals. These free radicals trigger enzymatic reactions, leading to oxidative damage to the cell membranes as well as the production of toxic metabolites and cell injury involving DNA, proteins, and lipids (63, 64).

Interestingly, the common factor between diabetes, as discussed in the previous section, and IRI is that OS affects the deterioration of the pathological processes, including inflammation. During IRI, the damaged tissues produce excessive amounts of ROS, causing the release of proinflammatory cytokines and apoptosis (64–66). After myocardial ischemia, cardiac surgery, cardiogenic shock, or circulatory arrest, myocardial IRI can lead to adverse cardiac events. Although it is necessary to restore the blood flow to nourish the cells, reperfusion is known for its harmful effects because of OS and the subsequent development of intense inflammation and immune responses (67–75). The following subsections discuss the role of the three molecules involved in the development of IRI.

### TLR4

Innate immune response to invading pathogens, which is derived from the toll receptors, is shared extensively among insects and vertebrates (76). Toll-like receptor 4 (TLR4) binds to various types of ligands such as lipopolysaccharides (LPS), low-density lipoproteins, and heat-shock proteins (77, 78). Among the toll-like receptors (TLRs) consisting of 11 subtypes in humans, TLR2 and TLR4, predominantly TLR4, are involved in the development of IRI (79). The TLR4-signaling pathway is an important inflammatory cascade in IRI with essential functions in the adaptive immune system (80, 81). Toll-like receptor 4 responds to endogenous molecules during the sterile inflammatory processes such as IRI (82) and is considered as the key regulator in several ischemia–reperfusion models.

As discussed earlier, OS is critically involved in the pathogenesis of IRI. In fact, ROS facilitates TLR4 trafficking to the plasma membrane, thereby promoting the TLR4 activity (83, 84). This event implies that the pathogenesis of IRI is at least partly attributable to the effect of ROS on the TLR4 activation. Furthermore, Pahwa et al. postulate that ROS act as a potential activator of TLRs and that hyperglycemia-induced OS activates TLRs, subsequently inducing inflammatory responses in diabetes (85).

The activations of TLR2, TLR3, and TLR4 increases oxidation levels of lipids and proteins (86). In addition to the TLR4 activation by ROS mentioned earlier, the relationship between ROS and TLR4 includes ROS production through the TLR4 activation. For example, TLR4 activation induced by LPS facilitate intracellular ROS production via NOX-4 (87). In TLR4-deficient mice, the ROS generation is reduced (88).

NF-κB initiates and disseminates innate immune responses by regulating the gene pools that encode proinflammatory/inflammatory cytokines (i.e., TNF-α, IL-1β, IL-6, and granulocyte/macrophage-colony stimulating factor), adhesion molecules (i.e., vascular cell adhesion molecule-1, intercellular adhesion molecule-1, and E-selectin), and chemokines (e.g., IL-8, regulated by the activation of normal T-cells expressed and secreted, MIP-1α, and MCP-1) (89, 90). The activation of TLR4, which forms a complex with several proteins such as CD14, myeloid differentiation primary response 88 (MyD88), and tumor necrosis factor receptor-associated factor 6 (TRAF6), leads to NF-κB activation (91–93). Reactive oxygen species acts on this TLR4/NF-κB pathway and further facilitates the NF-κB activation (94). Ischemia–reperfusion also leads to NF-κB activation (95).

The TLR4/NF-κB pathway is involved in the development of myocardial IRI. TLR4, initially detected in monocytes, is also expressed in other tissues, including the heart (76). Moreover, TLR4 is strongly expressed in injured myocardium (96). MAPKs, such as p38 and c-Jun NH2-terminal kinase (JNK), are activated during myocardial IRI (97), which in turn induces an acute inflammatory reaction. According to Lee et al., ROS produced by NOX-2/4 causes MAPK activation (98). TLR4-deficient mice have significantly less myocardial injury, as characterized by the reduction in the myocardial infarction area, decrease in the JNK and NF-κB activation, as well as reduction in the mRNA expression of inflammatory cytokines, such as IL-1β, IL-6, and MCP-1 (99).

The TLR4/NF-κB pathway is also involved in the development of IRI in other organs. The deletion of TLR4 or pharmacological antagonists reduces the severity of IRI in cardiac, hepatic, renal, and pulmonary models (99–108). In case of the lung IRI, the levels of phosphorylated JNK and NF-κB are diminished in TLR4-deficient mice (106, 108). Two pathways that possibly get activated during the lung IRI are apoptosis, induced by the activation of a transcriptional program controlled by NF-κB and acute inflammation promoted by the activation of resident alveolar macrophages and the expression of several proinflammatory cytokines and chemokines, such as TNF-α, IL-1β, IL-8, and macrophage inflammatory protein 2 (MIP-2) (109). The markers of lung injury, including permeability index, myeloperoxidase content, and bronchoalveolar lavage inflammatory cell counts were all decreased with TLR4 knockdown. The TLR4 knockdown in alveolar macrophages resulted in almost complete weakening of the lung IRI. The protective effect of TLR4 knockdown appears to be partly mediated by the significant reduction in pre-transcriptional signaling through MAPKs phosphorylation and possibly due to the nuclear translocation of transcription factors, such as NF-κB and activator protein-1 (107, 110).

### DPP4/CD26

Dipeptidyl peptidase-4 (DPP4), also known as CD26, is a cell-surface protease offers a wide range of biological functions. As a serine-type protease, DPP4 cleaves dipeptides from the N-terminus, with proline residues in the penultimate position (111, 112). Clinical and experimental study over the past 30 years has clearly demonstrated that the DPP4/CD26 pathway is involved in a variety of physiological processes and immune system diseases (113). In addition, DPP4/CD26 transmembrane glycoproteins are expressed not only by various cells of the immune system but also by the epithelial and systemic vascular endothelial cells, by the endothelial cells of venules and capillaries, by the cells of the heart, kidney, lung, pancreas, spleen, and small intestine, by the vascular smooth muscle cells, and by monocytes and hepatocytes; moreover, it is soluble in the plasma (111, 114, 115).

DPP4 lyses multiple peptide substrates, including the incretin hormone glucagon-like peptide-1 (GLP-1) (116). Glucagon-like peptide-1 inhibits OS generation and the subsequent inflammation (117–119). For example, GLP-1 exerts antioxidant effects via cyclic adenosine monophosphate (cAMP), phosphoinositide 3-kinase (PI3K), and protein kinase C-delta (PKCδ) pathways in diabetes (120). Dipeptidyl peptidase-4 inhibitors prolong the bioavailability of the endogenously secreted GLP-1, thereby exerting a beneficial therapeutic effect on diabetes (116, 121).

In addition to its involvement in the development of diabetes, accumulating evidence indicates the role of DPP4 in IRI (122). Dipeptidyl peptidase-4 deficiency preserves cardiac functions via GLP-1 signaling in myocardial IRI (123). In this regard, cardiomyocytes deficient in DPP4 are resistant to H_2_O_2_-induced cell death by activating the AKT signaling (124). Dipeptidyl peptidase-4 inhibitors reduce myocardial infarct size, improve the cardiac function, and promote the myocardial regeneration (125). The involvement of GLP-1 signaling in the preservation of cardiac functions has been confirmed in various animal model experiments, such as heart failure and myocardial infarction (123, 126–129). Glucagon-like peptide-1 inhibits apoptosis or necrosis of endothelial cells (118) and cardiomyocytes (130). Glucagon-like peptide-1-based therapies play an important role in the protection from myocardial IRI (127, 131–133).

The lung is the second-highest expressed organ of DDP4 in rats (134). Dipeptidyl peptidase-4 can directly affect the dynamics of lung inflammation and may itself act as a proinflammatory signaling molecule (135, 136). In the lung, the capillaries may act as the main source of DPP4 activity, while the submucosal serous gland and alveolar cells also express DPP4 (111). Similar to the case of myocardial IRI, GLP-1 is believed to exert a protective effect also in the lung IRI by suppressing the production of OS (137).

### HO-1

The presence of excessive free heme facilitates ROS formation, thereby leading to abnormal endothelial cell function, as observed in systemic hypertension, diabetes, and IRI (19384082). HO is important to reduce the production of ROS (138). Specifically, HO possesses the ability to degrade heme and produce carbon monoxide (CO), a heme ligand, and biliverdin, an antioxidant (139). Human HO exists in three isoforms, HO-1, HO-2, and HO-3. Among these, HO-1 is involved in exerting protective effect against IRI.

The expression of HO-1 is modulated by the transcription factor NRF2, as discussed in Section Obesity plays an important role in heart disease of diabetic patients. NRF2, which translocated to the nucleus under OS, activates antioxidant response element and increases the transcription of antioxidant genes, including HO-1 (140). The HO-1 system includes four main functions: (1) antioxidant function; (2) maintenance of microcirculation; (3) regulation of cell cycle; and (4) anti-inflammatory function (141). Overexpression of HO-1 exerts a potent cellular protective effect in rat heart ischemia–reperfusion models. HO-1 can reduce IRI due to the enhanced antioxidant and anti-apoptotic activities (142, 143).

Moreover, HO-1 possesses antiapoptotic outcomes. These effects get mediated through the p38 MAPK-signaling transduction pathway activated by CO (144). In addition, CO-exposed animals, at least partially, demonstrate a significant reduction in hyperoxia-induced lung apoptosis through the anti-inflammatory MKK3/P38 MAPK pathway (144). Three major MAPKs in cardiomyocytes are affected by the ischemia–reperfusion, and the ERK pathway may be critical for cell survival by protecting the cells from programmed cell death caused by stress-induced activation of p38 and JNK (145).

## Effects of ROS on the ION Channels and Their Implication With Pathophysiology

The transient receptor potential (TRP) melastatin (TRPM) subfamily belongs to the TRP cation channel superfamily, and most of its members either have calcium ion permeability or are calcium ion activating proteins (146, 147). Changes in the concentration of Ca^2+^/Mg^2+^ in cells or changes in the cell membrane potential and electrical activity can affect various biological processes, including the cellular OS level (148), endothelial cell permeability (149), and cell death (150). Therefore, in the past 10 years, the members of this family have attracted more and more interest and attention to CVD (151, 152), T2DM (153), and inflammation (154). The activity of some members of the TRPM subfamily is regulated by OS (155). Therefore, the emergence of OS-regulated ion channels in an oxidative environment creates favorable conditions for disease development.

### TRPM4 in Cardiomyocytes

TRPM4 is widely expressed in various tissues (156–159), including the atria and ventricles in both rodents (160, 161) and human (162, 163).

With the increase of OS, the TRPM4 channel functions abnormally, which promotes the onset and development of the disease. To verify this point, it became necessary to create an ischemic and hypoxic cellular environment. Presently, cobalt chloride (CoCl_2_) (164) and H_2_O_2_ (165, 166), in a laboratory setting, are widely used to establish OS models and fully characterized chemical agents. CoCl_2_ can be used to establish a simple *in vitro* model of hypoxic/ischemic disease in the laboratory, but up to now, there are few studies on TRPM4 channel induced by CoCl_2_. The possible reason is that CoCl_2_ can induce the production of ROSs, but also affect the expression of some genes, such as HIF-1α, p53, p21, and PCNA (167–169). CoCl_2_ may also affect the remodeling of CMs in hypoxic/ischemic area by activating PI3K/Akt and MAPK pathways (170), and CoCl_2_-induced apoptosis may be related to mitochondria-mediated apoptosis pathway (171). Hydrogen peroxide increases the activity of TRPM4 (172), while ATP and ADP inhibit its activity (173). When ATP production in hypoxia is insufficient, cardiomyocytes activates the K_ATP_ channels (174) and cause cell hyperpolarization, thereby preventing arrhythmia. However, this process may be affected by electrical disturbances induced by TRPM4 protein, because the channel is sensitive to Ca^2+^ and ATP (175, 176). Meanwhile, our previous research results (166) demonstrated that TRPM4 is involved in the death of cardiomyocytes mediated by H_2_O_2_. At higher concentrations, H_2_O_2_ increases cell death in a concentration-dependent manner, while 9-phenanthrol (9-Phe) can partially reverse H_2_O_2_-induced cell death. The reversal effect is probably the result of 9-Phe's direct effect on the TRPM4 channel (166, 177, 178).

### TRPM2

Unlike TRPM4, TRPM2 is a cation channel permeable to Ca^2+^ (179). TRPM2 also plays an important role in cell proliferation and survival (180). It is widely distributed and sensitive to OS (181). However, at present, there is little information available on the physiological and pathophysiological functions of TRPM2 in the heart. Early studies of the TRPM2 channel function support the observation that TRPM2 activation induces cell death by continuously increasing the [Ca^2+^]_i_ (182– 184).

Mitochondrial integrity is critical to the survival and function of cardiomyocytes and is essential for maintaining the high-energy requirements of cardiomyocytes. Ca^2+^ overload can lead to mitochondrial permeability transition (MPT), but Ca^2+^ overload is the result of bioenergy failure after MPT occurs following myocardial ischemia–reperfusion (185). This result can be corroborated from the study of Davidson et al. (186). In Langendorff-perfused mouse hearts, MitoQ, a mitochondrial-targeted scavenger of ROS, could significantly reduce the Ca^2+^ wave-related mPTP opening. The mitochondria can thus benefit from the calcium influx mediated by TRPM2 to reduce the mitochondrial ROS production (179).

The heart consumes an equivalent of 6 kg of ATP per day, most of which is produced through mitochondrial oxidative phosphorylation (187). Myocardial ischemia consumes a large amount of ATP and produces a large amount of ROS; this process reduces mitochondrial biogenesis and mitochondrial dysfunction, ultimately leading to cell death (39, 188). However, the results of a study showed (189) that TRPM2 can rescue the ATP levels in the cells. During OS, TRPM2 maintains cell survival after OS by regulating the antioxidant pathway and cofactors that are regulated by NRF2.

Moreover, the TRPM2 channels can protect cardiomyocytes from IRI (181), which may be due to the Ca^2+^ flux mediated by TRPM2 that enhances the activity of calcineurin and the stability of hypoxia-inducible factor (HIF) (190). In immune cells, the NOX activity depends on membrane depolarization (191) when the TRPM2 channel is activated and it inhibits the production of ROS. TRPM2-mediated calcium influx can reduce the production of ROS through the depolarization of the plasma membrane of immune cells and the negative feedback regulation of ROS production (192). This event contributes to cell functions such as cytokine production, insulin release, cell motility, and cell death (193).

### L-Type Voltage-Gated Calcium Channel

Pulmonary circulation is characterized by low resistance and low pressure, and the mean pulmonary arterial pressure (mPAP) is <20 mmHg (194). Hypoxic pulmonary vasoconstriction (HPV) is a physiological response of the arterioles. However, there is usually no obvious effect on the pulmonary arterial pressure during HPV on limiting the hypoxia area (195). Persistent hypoxia induces pulmonary vasoconstriction and vascular remodeling mediated by the contraction and proliferation of pulmonary artery smooth muscle cells (PASMC), which eventually led to pulmonary hypertension (PH) (196). Pulmonary hypertension associated with hypoxia belongs to the third group in the classification of PH (194). Although there is no unified view yet on this association, hypoxia could increase the level of ROS in PASMC (197–205).

Excessive ROS is considered to be the main factor of arterial remodeling in PH induced by chronic hypoxia (CH) (206, 207). The specific mechanism of ROS promoting PH has not been clarified yet, but it is evident that ROS plays an important role in CH-induced PH vasoconstriction. Abnormal voltage-dependent Ca^2+^ influx is considered to be related to the pathogenesis of hypoxic PH (HPH) (208). In PASMC, cytosolic Ca^2+^ concentration ([Ca^2+^]_cyt_) is regulated by two pathways: voltage-dependent Ca^2+^ influx and voltage-independent Ca^2+^ influx. The influx of Ca^2+^ through L-type voltage-gated calcium channels (VGCC) is an important [Ca^2+^]_cyt_ regulatory pathway in HPH. Nifedipine and verapamil, which are L-type VGCC antagonists, can prevent HPV, inhibit PASMC proliferation, and alleviate HPH (208–211). L-type VGCC belongs to one of the calcium ion channels, which is a polymer transmembrane protein complex composed of five subunits of α1, α2, δ, β, and γ. Here α1 is the main functional subunit, while the others are auxiliary subunits. There are four subtypes of α1: α1S (Ca_v_1.1), α1C (Ca_v_1.2), α1D (Ca_v_1.3), and α1F (Ca_v_1.4) (212). Ca_v_1.2 was upregulated, while L-type VGCC could functionally enhance pulmonary vasoconstriction associated with Ca^2+^ influx in PASMCs after CH exposure (213).

The existing pharmacological data indicates that L-type VGCC plays an important role in the increase of [Ca^2+^]_i_ in PASMC induced by acute O_2_ tension (214–218). Experiments are hence necessary to investigate the effects of specific inhibitors (such as mibefradil) of T-type VGCC to determine their role in maintaining [Ca^2+^]_i_ during hypoxia, although mounting evidence have demonstrated that the application of H_2_O_2_ (219– 221) and oxidized glutathione (GSSG) (222, 223) resulted in Ca^2+^ influx through L-type VGCC. In addition, the possibility of channel opening and inward Ca^2+^ currents are increased by Ca_v_1.2 subunit of L-type VGCC, which was glutathionylated by H_2_O_2_ and GSSG in subsequent studies (222, 223). Moreover, Ca^2+^ signaling contributed to the contraction of PA (224). Furthermore, L-type VGCC has been reported to be sensitive to plasma membrane depolarization (225). Interestingly, vasoconstrictor endothelin-1 (ET-1) can stimulate L-type VGCC-mediated increase of Ca^2+^ in PASMCs of CH Wistar rats through the PKC and Rho kinase-dependent ways (226, 227). This situation is not difficult to understand, because both PKC (228) and Rho kinase (229) can be activated by oxidation to regulate this process. An indirect evidence of this finding is that ET-1 could increase the production of ROS in PASMCs (230–232). This hypothesis has not been tested in pulmonary circulation, but the activation of L-type VGCC induced by ET-1 in isolated cardiomyocytes is now known to be mediated by.${\text{O}}_{2}^{-}$ (233).

## Conclusion

Oxidative stress is based on the balance between oxidant and antioxidant activities derived from numerous molecules and pathways. In this review, we discussed ROS production in hyperglycemia under diabetic conditions, and, interestingly, the effect of obesity on it. Moreover, OS affects calcium handling via SERCA2 and CaMKII, thereby exacerbating cardiac functions in diabetes. In this way, OS is involved in the effects of diabetes on CVD. Moreover, a common mechanism is involved in the pathology of diabetes and IRI. For example, the OS-induced inflammation basically shares the common mechanism of TLR4/NF-κB and TLR4/MAPK pathways in diabetes and IRI. In addition, the DPP4/GLP-1 and NRF2/HO-1 systems are involved in ROS scavenging in diabetes and IRI. We also discussed the effect of OS on the activities of ion channels, such as TRPM2, TRPM4, and L-type VGCC, and their implications with diseases, including IRI. Further understanding of these mechanisms is expected to promote the development of new strategies for the prevention and cure of these formidable diseases.

## Author Contributions

All authors listed have made a substantial, direct and intellectual contribution to the work, and approved it for publication.

## Conflict of Interest

The authors declare that the research was conducted in the absence of any commercial or financial relationships that could be construed as a potential conflict of interest.
